# Early and Late Complications of Mandibulectomy Free Flap Reconstruction: Does the Selective Use of Soft Tissue Only Flaps Reduce Complications?

**DOI:** 10.1177/00034894241250177

**Published:** 2024-04-27

**Authors:** Dylan B. McBee, Michael J. DiLeo, Caroline C. Keehn, Andrew T. Huang, Angela D. Haskins, David J. Hernandez

**Affiliations:** 1 Bobby R. Alford Department of Otolaryngology—Head and Neck Surgery, Baylor College of Medicine, Houston, TX, USA

**Keywords:** mandibular reconstruction, free tissue flaps, postoperative complications

## Abstract

**Purpose:**

This study aims to evaluate the factors most associated with early and late complications following microvascular free tissue transfer (MVFTT) after mandibulectomy.

**Methods:**

A retrospective review of patients undergoing MVFTT after segmental mandibulectomy from September 2016 to February 2021 was performed across a single academic institution. Surgical variables were collected, including the location of the resultant mandibular defect (anterior vs posterior) and flap type (osseous or non-osseous). The primary outcome variables included postoperative complications (early, <90 days; and late, >90 days) and the patients’ functional status (return to oral intake). Descriptive statistics, chi-square test, Fischer’s exact test, and 2-sample t tests were used to analyze differences among variables.

**Results:**

We analyzed a cohort of 114 consecutive patients with mandibular defects, comprising 57 anterior and 57 posterior defects. Bony free flaps with hardware were used to reconstruct 98% of anterior defects compared to 58% of posterior defects (*P* < .001). All soft tissue only flaps did not utilize any hardware during the reconstruction. Anterior defects demonstrated more late complications requiring additional surgery (30% vs 9%, *P* = .04). A secondary analysis of posterior mandibular reconstructions compared soft tissue only flaps and bony free flaps with hardware and showed equivalent rates of early (12% vs 13%, *P* > .99) and late (9% vs 8%, *P* > .99) complications requiring additional surgery while demonstrating a similar return to full oral competence (55% vs 46%, *P* = .52) and recovery of a 100% oral diet (67% vs 54%, *P* = .53).

**Conclusion:**

Osseous free tissue transfer for segmental mandibular defects remains the gold standard in reconstruction. In our patient cohort, anterior mandibular defects are associated with greater late (>90 day) complications requiring additional surgery. Comparable outcomes may be achieved with soft tissue only versus osseous free flap reconstruction of posterior mandibular defects.

## Introduction

The use of microvascular free tissue transfer (MVFTT) for mandibular reconstruction has become a common practice, especially after oncologic resections. In these cases, surgeons must balance patients’ cosmetic and functional desires while remaining cognizant of complications and adjuvant therapies that lay ahead. Despite these challenges, reconstructive efforts are essential for restoring oral competence, separating the aerodigestive tract from the neck, maintaining lower facial (mental) projection, and allowing for rehabilitation of swallowing. Reconstruction of anterior defects, or those involving the symphysis and surrounding bone, are particularly critical for maintaining lower facial projection. Anterior bony reconstruction improves oral intake and restores patency of the native airway.^1^ Socially, proper reconstruction may improve speech articulation and approximate the natural contour of the jaw—avoiding the Andy Gump deformity.^1^ While posterior defects lead to less dysfunction and disfigurement, bony reconstructive efforts diminish the degree of malocclusion and allow for integration of intraosseous dental implants.^2^

MVFTT remains a standard of care for many forms of mandibular reconstruction. The fibula and iliac crest provide adequate length and cortical density to reconstruct the mandible while allowing for osseointegrated dental restoration.^2-4^ Furthermore, the osseous radial forearm free flap may be elevated to satisfactorily reconstruct both anterior and lateral mandibular defects.^5^ When large, soft tissue defects accompany mandibular resections, chimeric or additional soft tissue flaps may be harvested. Likewise, a growing body of work demonstrates innovative techniques to reconstruct a range of mandibular defects using the scapula.^6,7^

Despite its preeminence, vascularized bone is not without intrinsic limitations. Bony flaps require hardware for fixation, which may lead to hardware exposure or infection, particularly after adjuvant radiation. These flaps also have a small risk of developing osteoradionecrosis secondary to adjuvant radiation.^8^ In select patients, donor site morbidity may be a concern due to loss of function at the donor site and/or slow return to ambulation.

Using soft-tissue free flaps to reconstruct posterior mandibular defects remains controversial. However, a select number of studies have demonstrated acceptable cosmetic and functional outcomes using soft tissue alone.^2,9-12^ As the conversation shifts to one in which there may be a time and a place for soft-tissue free flaps, we add to this debate. In the present study, we secondarily sought to compare both functional outcomes and complications for bony free flaps with hardware and soft-tissue only free flaps used for reconstructing the posterior mandible.

## Patients and Methods

A retrospective review of patients undergoing segmental mandibulectomy and reconstruction using MVFTT was conducted with institutional review board approval from Baylor College of Medicine. Data was collected from September 2016 to February 2021. Patients were included if they had undergone segmental mandibulectomy with subsequent MVFTT reconstruction. Patients were excluded if they had less than 90 days of follow up. All surgeries were performed using 2 surgical teams. All non-bony (soft tissue only) flaps did not utilize any hardware during the reconstruction. Collection and analysis of the current data was performed in a manner consistent with existing standards for clinical research (Declaration of Helsinki, US Federal Policy for the Protection of Human Subjects).

Patient characteristics including age, sex, BMI, smoking, and diabetic status as well as prior and postoperative chemotherapy or radiation treatment were collected for each subject. The Head and Neck Charlson Comorbidity Index (HN-CCI) was used to stage the comorbidity burden of each patient and serves as a validated prognostic tool for predicting survival in head and neck cancer patients.^13^ The HN-CCI was calculated for each patient according to their COPD, CHF, stroke, liver disease, and peptic ulcer disease status.

The indication for mandibulectomy and the site of cancer (if appropriate) were recorded. Defects in the mandible were categorized as either anterior or posterior. Anterior defects were defined as any segmental mandible defect that involved any of the symphysis between the canines, whereas posterior defects were segmental mandible defects that did not include any of the symphysis between the 2 canines. Using the Jewer classification system, hemi and lateral mandibulectomies would then be classified as posterior, while central, lateral-central, and lateral-central-lateral mandibulectomies would be classified as anterior. The Jewer classification also inherently defines the number of osteotomies required for the resection, with hemi mandibulectomies requiring 1 osteotomy and all others requiring 2. Flap characteristics were collected for all surgeries and included donor site, chimeric status, flap components, and hardware. Accompanying soft tissue resections were recorded and were categorized as those involving the lip, tongue, buccal mucosa, oropharynx, larynx or hypopharynx, and skin. Neck dissections and tracheostomy status for each surgery were also recorded.

Postoperatively, complications before and after 90 days were stratified according to type: fistula formation, head and neck infection, or hardware complication. Corresponding takeback surgeries were recorded while the ultimate flap outcome was reported as either a success, partial loss, or total loss. Finally, functional outcomes including tracheostomy decannulation, pre- and post-operative trismus, oral diet, and return to 100% PO were collected. Trismus was defined based on patient subjective complaint of difficulty opening their jaw. Full oral competence was determined on physical exam and was defined as the absence of anterior spillage or drool with demonstrated full labial closure and intact sucking ability.

Statistical analysis was performed using IBM SPSS Statistics for Mac OS, Version 28.0 (IBM Corp., Armonk, NY) and Stata/BE for Mac OS, Version 17.0 (StataCorp, College Station, TX). Continuous data were analyzed using the 2-sample *t* test. Frequency data were compared using the chi-square test. When the smallest expected value was less than or equal to 5, the Fischer’s exact test was used alternatively. A value of *P* < .05 was considered statistically significant. All p-values reported are 2 sided.

## Results

### Patient Characteristics

In this study, we analyzed a cohort of 114 consecutive patients with mandibular defects, consisting of 57 anterior and 57 posterior defects. Patient characteristics are summarized in Table 1. Patients who underwent reconstruction of the anterior mandible were significantly more likely to be former or active smokers (77% vs 59%, *P* = .04). They were also significantly older with a median of age of 65 compared to 61 (*P* = .02). Those undergoing posterior reconstruction were more likely to be male (86% vs 70%, *P* = .042) compared to a greater percentage of women undergoing anterior reconstructions. There was no significant difference in patient preoperative BMI between the anterior and posterior mandibular reconstruction groups. Similarly, there was no significant difference in prior or postoperative chemotherapy or head and neck radiation treatment between anterior and posterior mandibular reconstructions. Further, there was no significant difference in calculated HN-CCI (0.44 anterior vs 0.42 posterior, *P* = .90). Additionally, no difference was appreciated in any individual comorbidity between the 2 groups. The average length of follow up for each patient was 16 ± 2.2 months.

**Table 1.** Patient Characteristics by Mandibular Defect.

Characteristics	Anterior (n = 57)	Posterior (n = 57)	*P*-value
Sex (males)	**40**	**49**	**.042** ^a^
(Females)	**17**	**8**	
Median age (y)	**65**±**2**	**61**±**3**	**.02** ^c^
Mean BMI	25.0±5.1	25.1±5.5	.89^c^
Underweight BMI	7	6	.77^a^
Overweight/obese BMI	27	26	.85^a^
Prior chemotherapy	12	17	.28^a^
Prior HN radiation	18	18	>.99^a^
Post-operative chemotherapy	19	13	.21^c^
Post-operative HN radiation	28	25	.57^c^
Diabetes	6	4	.74^b^
Smoker (active or former)	**44**	**34**	**0.04** ^a^
CHF	1	1	>.99^b^
COPD	4	2	.68^b^
CVA	1	3	.62^b^
Liver disease	6	5	>.99^b^
Peptic ulcer disease	2	3	>.99^b^
HN-CCI	0.44±0.18	0.42±0.21	.90^c^

a χ^2^ test.

b Fisher’s exact test.

c 2-sample *t* test.

Bold values represent significant differences.

### Flap Characteristics and Perioperative Outcomes

The primary indication for flap surgery varied among mandibulectomy patients: cancer (82), osteoradionecrosis (20), benign tumors (4), post-treatment reconstructions (3), trauma (2), and bisphosphonate osteonecrosis (3). Malignancies were stratified according to the location of the primary tumor: oral cavity (62), oropharynx (10), skin (4), salivary gland (4), other (2).

Table 2 presents flap characteristics and perioperative outcomes by the type of reconstruction. Anterior defects of the mandible were significantly more likely to be reconstructed with a vascularized bony flap and accompanying hardware (98% vs 2%, *P* < .001). In contrast, posterior defects were much more likely to be reconstructed with soft tissue alone (42% vs 58% bone, *P* < .001). Neither group demonstrated significantly more flaps that required a chimeric architecture. More granularly, anterior reconstructions were more likely to utilize the fibular (*P* = .01) and scapular (*P* = .001) free flap donor site, while posterior reconstruction were more likely to use the anterolateral thigh free flap (*P* < .001). Importantly, no difference was appreciated between anterior and posterior defects in terms of the soft tissue components resected. That is, no difference between intraoperative lip, buccal mucosa, oropharynx, tongue, skin, larynx, or hypopharynx resection was found. Similarly, anterior and posterior reconstructions had equivalent rates of neck dissection. The length of surgery for reconstruction of the anterior mandible was significantly longer (13.1 vs 11.9 hours, *P* = .02). Yet there no was no significant difference in the length of postoperative hospital stay (11.5 days anterior vs 10.6 days posterior, *P* = .35).

**Table 2.** Perioperative Outcomes by Mandibular Defect.

Perioperative outcomes	Anterior (n = 57)	Posterior (n = 57)	*P*-value
Jewer (HCL) classification			
Hemi mandibulectomy	NA	37	NA
Central mandibulectomy	6	NA	NA
Lateral mandibulectomy	NA	20	NA
Central-lateral mandibulectomy	32	NA	NA
Lateral-central-lateral mandibulectomy	19	NA	NA
Flap type			
Bone	56	33	<.001^a^
Soft tissue	1	24	<.001^b^
Chimeric	21	16	.32^a^
Flap site			
Fibula	44	31	.01^a^
Anterolateral thigh	3	25	<.001^a^
Scapular	12	1	.001^a^
Pectoralis	0	1	>.99^b^
Vastus lateralis	0	4	.12^b^
Resections	n = 48	n = 54	
Lip	6	5	>.99^b^
Buccal	14	25	.08^a^
Oropharynx	12	17	.47^a^
Tongue	19	12	.06^a^
Skin	11	5	.18^b^
Larynx/hypopharynx	1	1	>.99^b^
Neck dissection	40	46	.80^a^
Length of stay (d)	11.5±1.4	10.6±1.3	.35^c^
Mean surgery length (h)	**13.1**±**0.8**	**11.9**±**0.7**	**.02** ^c^

a χ^2^ test.

b Fisher’s exact test.

c 2-sample *t* test.

Bold values represent significant differences.

### Postoperative Complications and Functional Status

Postoperative complications and functional status according to the type of reconstruction are summarized in Table 3. While no difference in OR takeback was appreciated between the 2 groups in the first 90 days, complications requiring additional surgery thereafter were significantly greater for anterior defects (30% vs 9%, *P* = .04). Delayed hardware complications due to exposure or infection primarily drove additional surgeries for either cohort. While not significant, anterior free flaps were more likely to be complicated by osteoradionecrosis. It is worth noting that not every delayed complication required additional surgery. Of those that did, hardware extrusion or failure accounted for 53% of corrective surgeries for anterior defects and 80% of corrective surgeries for posterior defects. Overall, the failure rate for free flaps between anterior and posterior reconstructions was equivalent (3.5% vs 5.2%, *P* > .99). Of the 114 free flaps tracked, 2 flaps were completely lost and 3 were deemed a partial loss. There were no significant differences in pre- or post-operative trismus between the groups. Additionally, both cohorts returned to some form of an oral diet at similar rates (68% anterior vs 77% posterior, *P* = .29). However, multiple postoperative differences in functional status were found to favor those that underwent posterior mandibulectomy. Patients requiring anterior reconstruction required tracheostomy placement at greater rates (93% vs 79%, *P* = .03). Those patients with posterior defects were significantly more likely to return a 100% oral diet (61% vs 32%, *P* = .01). Further, those same patients were more likely to demonstrate full oral competence at their most recent postoperative office visit (51% vs 30%, *P* = .02).

**Table 3.** Postoperative Outcomes by Mandibular Defect.

Postoperative outcomes	Anterior (n = 57)	Posterior (n = 57)	*P*-value
OR takeback <90 d	6	7	.77^a^
Surgery >90 d	**17**	**5**	**.04** ^b^
Hardware complication	10	4	.15^b^
Soft tissue infection/fistula	4	2	.44^b^
Osteoradionecrosis	6	1	.11^b^
Flap failure (combined)	2	3	>.99^b^
Partial flap failure	1	2	>.99^b^
Total flap failure	1	1	>.99^b^
Functional outcomes			
Tracheostomy (n = 98)	**53**	**45**	**0.03** ^a^
Trach decannulation (n = 98)	39	40	.06^a^
Permanent trach (n = 98)	14	5	.07^b^
Trismus pre-operative	12	19	.14^a^
Trismus post-operative	10	18	.08^a^
Recovery of oral diet	39	44	.29^a^
100% PO	**18**	**35**	**.01** ^a^
Full oral competence	**17**	**29**	**.02** ^a^

a χ^2^ test.

b Fisher’s exact test.

Bold values represent significant differences.

### Postoperative Complications and Functional Status for Posterior Reconstruction

Table 4 highlights important complications and postoperative outcomes for posterior defects according to the type of free flap used. Vascularized bony flaps containing hardware and soft tissue only flaps showed equivalent rates of OR takeback within 90 days (12% vs 13%, *P* > .99) and additional surgery thereafter (9% vs 8%, *P* > .99). Additionally, rates of overall flap failure were equivalent (6% bone vs 4% soft tissue, *P* > .99). While a return to full oral competence was greater in osseous free flaps with hardware compared to soft tissue flaps alone, this difference was not statistically significant (55% vs 46%, *P* = .52). Further, recovery of any oral diet (82% bone vs 71% soft tissue, *P* = .33) as well as a return to 100% PO type (67% bone vs 54% soft tissue, *P* = .34) was not found to be significantly different according to flap type.

**Table 4.** Postoperative Outcomes for Posterior Mandibular Defects.

Postoperative outcomes	Osseous with hardware (n = 33)	Soft tissue only (n = 24)	*P* value
OR takeback <90 d	4	3	>.99^a^
Flap failure (combined)	2	1	>.99^a^
Additional surgery >90 d	3	2	>.99^a^
Full oral competence	18	11	.52^b^
100% PO	22	13	.34^b^
Recovery of oral diet	27	17	.33^b^

a Fisher’s exact test.

b χ^2^ test.

## Discussion

While frequently used in the reconstruction of mandibular defects, our study demonstrates that osseous flaps increase the possibility of late complications for a variety of diagnoses. Anterior, predominately bony reconstructions have an increased risk of returning to the operating room after 90 days. Factors inherent to most bony flaps including hardware complications and osteoradionecrosis primarily drove reoperations. Further, the most common cause of reoperations among posterior reconstructions was also hardware extrusion or failure.

Anterior defects are technically more challenging and lead to worse functional outcomes. Overall, anterior resections were much less likely to regain full oral competence or return to a 100% PO diet. This is in line with a large body of work demonstrating a return to preoperative oral function in only a fraction of patients.^14-16^ We recognize that the near selective use of bony free flaps remains most favorable for restoring facial projection and oral function in anterior defects. However, matching cephalometric dimensions of the native anterior mandible with transferred bone simply remains more challenging than in the lateral jaw. Thus, despite advances in surgical technique and postoperative management, anterior reconstruction continues to pose a challenge for surgeons and their patients. Patients with poor functional status are also less likely to tolerate longer operations seen with anterior repair. We know that lasting functional limitations or cosmetic deformity have particularly profound effects on patient well-being.^1^ Warshavsky et al^1^ point out that anterior resections have a greater impact on health-related and social quality of life domains when compared to their posterior counterpart. It should be noted that our patients with anterior resections were older and more likely to smoke when compared to those with posterior defects. Yet, smoking status has been shown to have a surprisingly modest effect on the overall outcomes of microsurgical free flap reconstruction.^17^ Similarly, advanced age alone provides less value for predicting functional outcomes than comorbidities, which were equal between groups.^18^

Posterior reconstruction of the mandible using vascularized bone flaps may provide the benefit of improved dental rehabilitation, occlusion, and potentially cosmesis. Although a return to full oral competence and a 100% PO diet was favored among osseous reconstructions, no functional outcome was significantly better when compared to soft tissue only flaps in this series. Hanasono et al^2^ found similar results suggesting that soft-tissue flaps can provide satisfactory dietary and mouth opening outcomes. They further acknowledge that soft-tissue free flaps can provide reasonable cosmetic results for more limited defects of the posterior mandible.^2^ A study of mandibular free flap reconstruction in the elderly argues that soft tissue flaps are indicated for posterior mandibular defects in those patients older than 75 years of age and those with poor dental occlusion.^9^

Soft tissue free flaps should be considered for posterior mandibular reconstruction, but only in the appropriate context. Those undergoing adjuvant radiation or bisphosphonate therapy may avoid unnecessary osteonecrosis with a soft tissue free flap.^8^ Further, in patients where osteointegrated dental implants are contraindicated or not feasible, vascularized bone provides a less distinct advantage. In patients with limited functional status, harvest of a bony flap (particularly the fibula free flap) may not be well tolerated due to the potentially prolonged OR time and additional functional limitation (slow mobility) post-operatively. Comorbidities including peripheral vascular disease can also limit possible donor site options eliminating ideal bony targets like the fibula.^19^ It must be noted that if soft tissue reconstruction is performed with the use of hardware, numerous complications may occur. Wei et al^20^ found that nearly 70% of their patients undergoing free flap reconstruction of composite mandibular defects using soft tissue free flaps experienced plate complications, most commonly plate exposure, occurring in 46% of patients. Another study found that 30.7% of their patients undergoing soft tissue only reconstruction of mandibular defects experienced plate failure, compared to 4.8% in those that received an osteocutaneous free flap.^21^

Modified or reduced hardware may limit late complications while maintaining the advantages of bony reconstruction. A recent study from Davies et al^22^ demonstrated that optimizing contouring of reconstruction plates to bone reduces the odds of plate exposure. Fujiki et al^23^ suggest a “no-touch-technique” for mandibular plates that prevents contamination by saliva and leads to significantly lower rates of plate-related infection and fistula formation. One alternative to the fibula free flap is the lateral scapular border or scapular tip free flap, which allows for more robust soft tissue coverage of hardware compared to the skin and soft tissue of the lower extremity.^7^ The subscapular system provides adequate soft tissue and bone with excellent 3-dimensional spatial mobility to reconstruct composite defects of the anterior and posterior mandible.^7^ Horizontal orientation of the scapular tip approximates the natural contour of the mandible and reduces the need for osteotomies.^7^ Importantly, harvesting scapular bone is associated with limited donor site morbidity and is less susceptible to peripheral vascular disease.^19,24^

The present study faces certain limitations. First, in the setting of a nonrandomized retrospective review, flap type was a tailored choice for each patient and their defect. While overall functional status, adjuvant therapies, and defect location were important considerations, surgeon preference also played a significant role in the type of flap used for reconstruction. Other factors such as dentition status played a role when deciding between bony versus soft tissue reconstruction specifically for posterior defects, along with prior comorbidities and radiation treatment. Furthermore, flap selection and harvest site follow regional and institutional trends. For example, while the osteocutaneous forearm flap may be used for mandibular reconstruction, this is not routinely done at our institution. Second, comparing functional outcomes and complication rates for anterior and posterior defects is inherently difficult. A strong preference for reconstructing defects that extend anteriorly with bone made comparison of anterior and posterior defects less clear. That is, we must be cognizant that a comparison of the 2 is influenced by the location of the defect and varying flap type. Third, our study was limited by the length of follow-up. One concern may be that our study provided inadequate time to capture all hardware failures. After all, Almansoori et al^25^ demonstrate that just 54.5% of plate fractures occurred within the first 2 years in a study that followed patients for 97 ± 5.4 months. However, this was only true for reconstruction plates used alone or with soft tissue flaps as 100% of plates used with bony flap reconstructions remained intact in this study.^25^ Our use of reconstruction plates was limited to bony free flaps and thus the risk associated with using hardware without a bony flap was eliminated in our study. Finally, a larger sample would have allowed for a more meaningful comparison of bone and soft tissue flaps for the reconstruction of posterior mandibular defects.

## Conclusion

Osseous flaps remain the gold standard in segmental mandibular reconstruction, but they may be associated with an increased risk of late complications, often related to hardware extrusion or failure. Defect location and the choice of reconstructive flap components, bony versus soft tissue, also contribute to these risks, with anterior defects being associated with more complications. In some cases, such as when feasible for posterior mandibular defects in edentulous patients, soft tissue reconstruction can provide adequate vascularized tissue, avoid many late complications, and allow for equivalent oral and swallowing rehabilitation. It is important to consider the risks and benefits of each reconstruction method to determine the most appropriate approach for each patient.
